# The impact of triglyceride-glucose index on incident cardiovascular events during 16 years of follow-up: Tehran Lipid and Glucose Study

**DOI:** 10.1186/s12933-020-01121-5

**Published:** 2020-09-29

**Authors:** Niloofar Barzegar, Maryam Tohidi, Mitra Hasheminia, Fereidoun Azizi, Farzad Hadaegh

**Affiliations:** 1 Prevention of Metabolic Disorders Research Center, Research Institute for Endocrine Sciences, Shahid Beheshti University of Medical Sciences, No. 24 Aarabi St. Velenjak, P.O. Box: 19395-4763, Tehran, Iran; 2 Endocrine Research Center, Research Institute for Endocrine Sciences, Shahid Beheshti University of Medical Sciences, Tehran, Iran

**Keywords:** Cardiovascular disease, Coronary heart disease, TyG-index

## Abstract

**Background:**

To investigate whether the Triglyceride-Glucose index (TyG-index) is associated with increased risk of cardiovascular diseases (CVD)/coronary heart disease (CHD).

**Methods:**

A total of 7521 Iranians aged ≥ 30 years (male = 3367) were included in the study. Multivariate Cox regression analyses (adjusted for age, gender, waist circumference, body mass index, educational level, smoking status, physical activity, family history of CVD, type 2 diabetes, hypertension, low and high density lipoprotein cholesterol, and lipid lowering drugs) were used to assess the risk of incident CVD/CHD across quintiles and for 1-standard deviation (SD) increase in the TyG-index. The cut off point for TyG-index was assessed by the minimum value of $\sqrt {\left( {1 - sensitivity} \right)^{2} + \left( {1 - specificity} \right)^{2} }$. We also examined the added value of the TyG-index in addition to the Framingham risk score when predicting CVD.

**Results:**

During follow-up, 1084 cases of CVD (male = 634) were recorded. We found a significant trend of TyG-index for incident CVD/CHD in multivariate analysis (both Ps for tend ≤ 0.002). Moreover, a 1-SD increase in TyG-index was associated with significant risk of CVD/CHD in multivariate analysis [1.16 (1.07–1.25) and 1.19 (1.10–1.29), respectively]. The cut-off value of TyG-index for incident CVD was 9.03 (59.2% sensitivity and 63.2% specificity); the corresponding value of TyG-index for incident CHD was 9.03 (60.0% sensitivity and 62.8% specificity), respectively. Although no interaction was found between gender and TyG-index for CVD/CHD in multivariate analysis (both Ps for interaction > 0.085), the significant trend of TyG-index was observed only among females for incident CVD (P = 0.035). A significant interaction was found between age groups (i.e. ≥ 60 vs < 60 years) and TyG-index for CVD outcomes in the multivariate model (P-value for interaction = 0.046). Accordingly, a significant association between the TyG-index and outcomes was found only among the younger age group. Among the population aged < 60 the addition of TyG-index to the Framingham risk score (FRS) did not show improvement in the predictive ability of the FRS, using integrated discrimination improvement.

**Conclusion:**

The TyG-index is significantly associated with increased risk of CVD/CHD incidence; this issue was more prominent among the younger population. However, adding TyG-index to FRS does not provide better risk prediction for CVD.

## Background

Over the past several years, cardiovascular diseases (CVD), including ischemic heart disease, heart failure, stroke, and several other cardiac and vascular conditions have played an important role in the global rate of mortality [1]. As with the rest of the world, CVD is the primary cause of morbidity and mortality in the Middle East and North Africa (MENA) [2]. It has been reported that about 80% of global CVD mortality is attributable to middle- and low-income countries where the burden of CVD and CVD risk factors are on the rise [3]. This fact makes it vital to recognize and control potential risk factors.

Insulin resistance (IR) is known to be one of the most important risk factors for CVD [4]. Not only is IR associated with CVD risk factors such as type 2 diabetes (T2D), hypertension, dyslipidemia, and obesity, but it is also an independent risk factor for CVD [5]. In this regard, Triglyceride-Glucose index (TyG-index), a product of triglyceride (TG) and fasting plasma glucose (FPG), is evaluated as a surrogate for IR [6–8]. Some studies have shown an association between TyG-index and incident T2D [9–12]. Cohort studies conducted among American, European, and Chinese populations have found increasing TyG-index level as an independent risk factor for incident CVD [13–17].

Due to the high burden of IR and CVD among residents of the MENA region [18], we examined the association between TyG-index and incident CVD and coronary heart disease (CHD) for the first time, during a follow-up of more than a decade in the oldest cohort of the region namely the Tehran Lipid and Glucose Study (TLGS). Moreover, we also examined these associations in each gender, and among elderly vs younger adult populations. Appropriate cut off points were also derived for this index in prediction of CVD/CHD events. Finally, the added value of TyG-index when included in the Framingham risk score (FRS) [19] in prediction of CVD was also examined.

## Methods

### Study population

This study was conducted within the framework of TLGS. TLGS is an ongoing large-scale community-based cohort study with a long-term follow-up. The study was initiated in 1999–2002 to estimate the prevalence of non-communicable diseases and related risk factors. Detailed descriptions and study design of TLGS have been reported elsewhere [20].

In brief, TLGS includes 15,005 participants at first visit (1999–2002), with an additional 3550 recruitments in the second visit (2002–2005). Follow-up visits are performed at approximately 3-year intervals. Participants of the TLGS aged ≥ 30 years (n = 9554) were included in this study (7928 from the first phase and 1626 from the second phase). After exclusion of subjects with prevalent CVD at baseline (n = 602), those with missing data for covariates including anthropometric measures, smoking status, T2D, hypertension, family history of CVD, and lipid measures (n = 749, considering overlap features between numbers), and finally those without any follow-up after baseline recruitment (n = 682), 7521 individuals remained in the study and were followed until March 2016.

This study was approved by the Ethics Committee of the Research Institute for Endocrine Science, Shahid Beheshti University of Medical Sciences, and all participants provided written informed consent.

### Clinical, anthropometric, and laboratory measurements

Participants were interviewed individually by trained interviewers. A standard pre-tested questionnaire was used to collect demographic information. Subjects were questioned about their physical activity, smoking status, level of education, drug history, past medical history, and family history. During the first phase of TLGS, physical activity pattern was assessed by the Lipid Research Clinic (LRC) questionnaire. As a result of the insufficient accuracy of LRC [21], it was replaced by the Modifiable Activity Questionnaire (MAQ) for future follow-up examinations [22]. Details of anthropometric measurements including weight, height, and waist circumference (WC) are reported elsewhere [20]. Body mass index (BMI) was calculated by dividing weight (kg) by squared height (m^2^). After 15 min rest in a sitting position, a physician measured blood pressure using a calibrated mercury sphygmomanometer. Systolic blood pressure (SBP) and diastolic blood pressure (DBP) were measured twice with at least 30 min interval and the mean value was considered as the participant’s blood pressure.

Blood samples were drawn between 7:00 and 9:00 am after 12 to 14 h of overnight fasting. Samples were centrifuged within 30–45 min of collection. Details of laboratory measures including total cholesterol (TC), TG, high-density lipoprotein cholesterol (HDL-C), FPG, and 2 h plasma glucose (2hPG) are reported elsewhere [20, 23]. Low-density lipoprotein cholesterol (LDL-C) was calculated from serum TC, HDL-C, and TG concentrations expressed in (mg/dL) using the Friedewald formula [24].

### Assessment and definition of the outcome

Follow-up for any medical outcome was repeated annually by phone call by a trained nurse. For medical conditions leading to hospitalization or mortality, a trained physician collected complementary data through a home visit and hospital documents [25]. Collected data were evaluated by an outcome committee consisting of an internist, endocrinologist, cardiologist, epidemiologist, pathologist and other experts when needed, in order to assign a specific outcome to every event. It is important to note that the outcome committee was blinded to the status of baseline risk factors.

In the current study, CHD was defined as any definite myocardial infarction (MI) diagnosed by electrocardiogram (ECG) and biomarkers (including troponin CK, CK-MB, CK-MBm, and myoglobin), probable MI (positive ECG findings plus cardiac symptoms and missing biomarkers or positive ECG findings plus equivocal biomarkers), unstable angina pectoris (new cardiac symptoms or changes in the symptom pattern, and positive ECG findings with normal biomarker values), and angiography proven CHD. CVD was determined as a composite of CHD plus fatal and non-fatal stroke.

### Definition of terms

The TyG-index was calculated as ln [fasting TG (mg/dL) × FPG (mg/dL)/2] [26]. The categorization of the TyG-index among the total population was as follows: quintile 1 (< 8.4) (as reference), quintile 2 (8.4–8.7), quintile 3 (8.7–9.0), quintile 4 (9.0–9.4), and quintile 5 (> 9.4). Hypertension was specified as SBP ≥ 140 mmHg or DBP ≥ 90 mmHg or the use of antihypertensive drugs. T2D was ascertained among participants who had FPG ≥ 7 mmol/L, 2hPG ≥ 11.1 mmol/L, or current use of anti-diabetic medication. We divided educational status into three groups: illiterate and < 6 years of education (as reference), 6–12 years, and ≥ 12 years which can be considered as primary, secondary and superior levels of education, respectively. Low physical activity was defined as exercising less than 3 days per week (according to LRC questionnaire) [21] and scores ≤ 600 MET (metabolic equivalent task)-minutes per week (according to MAQ questionnaire) [27] for participants who entered the study at the first and second phases of TLGS, respectively. A current smoker was defined as a person who smokes cigarettes, pipe or hookah daily or occasionally; those who were not currently smokers with a previous history of smoking were called past smokers; and those without any history of smoking were called never smokers (as a reference group).

### Statistical analysis

Continuous variables were expressed as the mean ± standard deviation (SD). Categorical variables are presented as frequency and percentage. We used one-way ANOVA, and Chi-squared test to compare the baseline characteristics of subgroups across TyG-index quintiles.

The cumulative incidence of CVD/CHD were calculated by dividing the number of cases by the number of subjects followed for each TyG-index category.

Since there were no interactions between gender and TyG-index for CVD/CHD in multivariate analysis (both Ps for interaction > 0.085), the analysis was performed among the entire population. However, to be comparable with other studies, we also performed data analysis in each gender. Multivariate Cox proportional-hazard analyses were performed to estimate hazard ratios (HRs) and 95% confidence intervals (CIs) of incident CVD/CHD. TyG-index was examined both across quintiles and for a 1-standard deviation (SD) increase. All analyses were adjusted for potential CVD/CHD confounders including age, gender (for total population), WC, BMI, educational level, smoking status, physical activity, family history of CVD, T2D, hypertension, LDL-C, HDL-C, and lipid lowering drugs based on biological plausibility and identification as a confounder in our previous studies [28]. We examined the variance inflation factor (VIF) of the variables included in the model to address the issue of collinearity [29]. We did not find evidence of collinearity in the model, given the VIF of < 5.

To determine the optimum cut off value for TyG-index in cases of incident CVD/CHD, we used the receiver operating characteristic (ROC) curve analysis. The best cut off point for TyG-index was assessed by the minimum value of $\sqrt {\left( {1 - sensitivity} \right)^{2} + \left( {1 - specificity} \right)^{2} }$, which represented the maximum sum of sensitivity and specificity.

Since we found significant interaction between age groups (i.e. ≥ 60 vs < 60 years) and TyG-index for CVD outcomes in the multivariate model (P for interaction = 0.046), we performed analysis according to age groups.

Finally, we fitted a prediction model based on variables used in the FRS including age, gender, TC, HDL-C, SBP, anti-hypertensive drugs, smoking, and T2D. Afterwards we developed a new model comprising the Framingham variables with the addition of baseline TyG-index. In this regard, we used Harrell’s concordance statistic (C-index) to evaluate discrimination of the predicting models (FRS model with and without TyG-index) and integrated discrimination improvement (IDI) as a criterion for evaluating the ability of TyG-index to improve CVD risk prediction over FRS [30, 31].

The proportional hazard assumption in the Cox models was assessed with the Schoenfild residual test; all proportionality assumptions were appropriate. Statistical analysis was performed using SPSS for windows version 20 and STATA version 14. A P-value of ≤ 0.05 was considered to indicate statistical significance.

## Results

### Baseline characteristics

Overall, a total of 7521 subjects (male = 3367) were included in the analysis. Baseline characteristics of the study population according to TyG-index quintiles are presented in Table 1. Generally, compared to the lowest quintile of TyG-index, those in the highest quintile were older, less likely to be female, had higher values of WC and BMI, were less educated and less physically active. Moreover, the prevalence of T2D, hypertension, and the use of lipid lowering drugs as well as baseline values of LDL-C were higher, whereas the value of HDL-C was lower among participants in the fifth quintile of the TyG-index.

**Table 1:** Baseline characteristics of the study population by quintiles of TyG-index: Tehran Lipid and Glucose Study

Variables	TyG-index quartiles					P-value	Total (n = 7521)
	1 (< 8.4)	2 (8.4–8.7)	3 (8.7–9.0)	4 (9.0–9.4)	5 (≥ 9.4)		
	(n = 1503)	(n = 1506)	(n = 1504)	(n = 1504)	(n = 1504)		
Age (years)	43.1 ± 12.3	45.5 ± 12.2	47.1 ± 12.0	47.3 ± 11.7	50.3 ± 11.4	< 0.001	46.6 ± 12.1
Gender							
Male	588 (39.1)	664 (44.1)	696 (46.3)	696 (46.3)	723 (48.1)	< 0.001	3367 (44.8)
Female	915 (60.9)	842 (55.9)	808 (53.7)	808 (53.7)	781 (51.9)		4154 (55.2)
WC (cm)	83.2 ± 10.7	88.3 ± 10.9	91.7 ± 10.3	94.0 ± 10.6	96.5 ± 10.2	< 0.001	90.7 ± 11.5
BMI (kg/m^2^)	25.1 ± 4.27	26.8 ± 4.39	27.9 ± 4.40	28.7 ± 4.32	29.1 ± 4.32	< 0.001	27.5 ± 4.57
Education (years)							
< 6	451 (30.0)	560 (37.2)	604 (40.2)	639 (42.5)	789 (52.5)	< 0.001	3043 (40.5)
6–12	834 (55.5)	749 (49.7)	708 (47.1)	698 (46.4)	559 (37.2)		3548 (47.2)
≥ 12	218 (14.5)	197 (13.1)	192 (12.8)	167 (11.1)	156 (10.4)		930 (12.4)
Smoking							
Never	1164 (77.4)	1118 (74.2)	1134 (75.4)	1118 (74.3)	1101 (73.2)	0.075	5635 (74.9)
Past	104 (6.9)	114 (7.6)	122 (8.1)	140 (9.3)	142 (9.4)		622 (8.3)
Current	235 (15.6)	274 (18.2)	248 (16.5)	246 (16.4)	261 (17.4)		1264 (16.8)
Low physical activity	1002 (66.7)	996 (66.1)	1066 (70.9)	1056 (70.2)	1071 (71.2)	0.002	5191 (69.0)
FH-CVD	227 (15.1)	239(15.9)	239 (15.9)	253 (16.8)	264 (17.6)	0.407	1222 (16.2)
T2D	16 (1.1)	54 (3.6)	78 (5.2)	195 (13.0)	645 (42.9)	< 0.001	988 (13.1)
Hypertension	162 (10.8)	281 (18.7)	375 (24.9)	464 (30.9)	577 (38.4)	< 0.001	1859 (24.7)
Lipid Lowering drugs	6 (0.4)	18 (1.2)	41 (2.7)	54 (3.6)	154 (10.2)	< 0.001	273 (3.6)
LDL-C (mmol/L)	2.98 ± 0.76	3.40 ± 0.81	3.61 ± 0.83	3.76 ± 0.92	3.91 ± 1.06	< 0.001	3.53 ± 0.94
HDL-C (mmol/L)	1.23 ± 0.29	1.13 ± 0.29	1.05 ± 0.26	1.01 ± 0.24	0.95 ± 0.25	< 0.001	1.07 ± 0.28

Data are represented as mean ± SD or frequency (percent)

*TyG-index* triglyceride and glucose index, *WC* waist circumference, *BMI* body mass index, *FH-CVD* family history of cardiovascular disease, *T2D* type 2 diabetes, *LDL-C* low-density lipoprotein cholesterol, *HDL-C* high-density lipoprotein cholesterol

After a median follow-up of 16.1 years (interquartile range: 13.4–16.5), 1084 (male = 634) and 924 (male = 542) cases of CVD and CHD occurred.

### TyG-index as a categorical variable

**Fig. 1:**
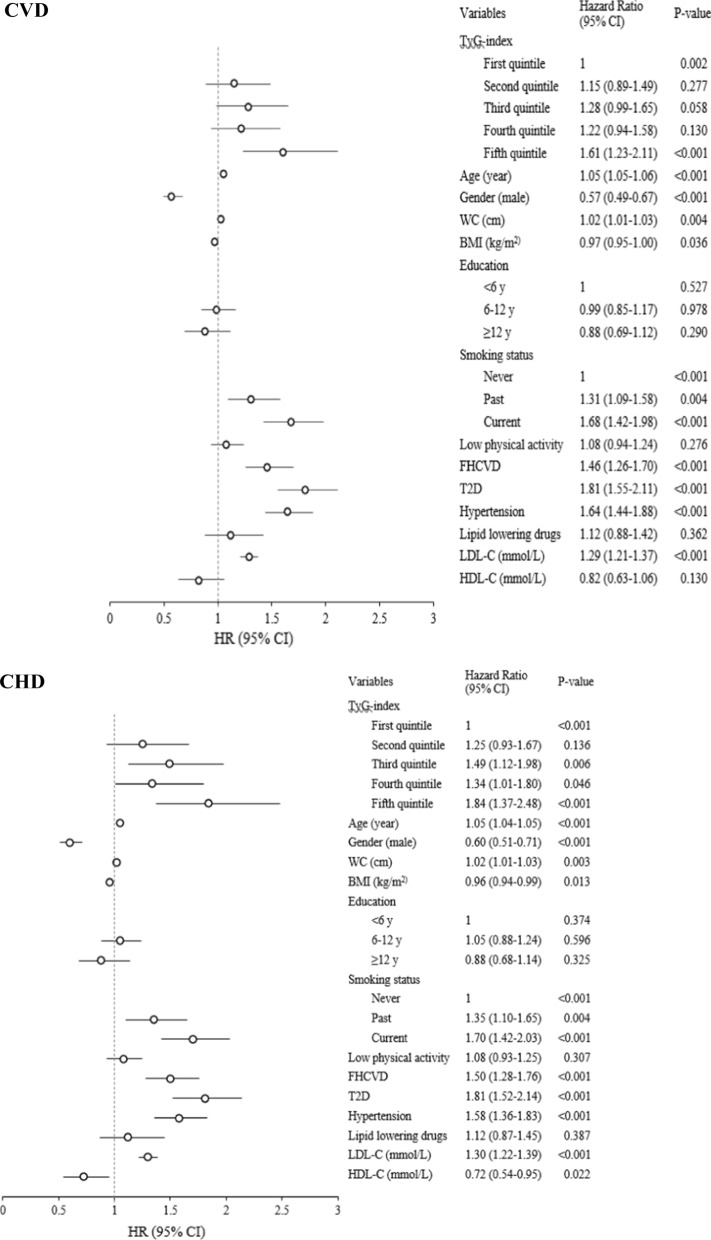
HRs (95% CI) of incident CVD and CHD for covariates. HR: hazard ratio; CI: confidence interval; CVD: cardiovascular disease; CHD: coronary heart disease; TyG: triglyceride and glucose index; WC: waist circumference; BMI: body mass index; FHCVD: family history of CVD; T2D: type 2 diabetes; LDL-C: low-density lipoprotein cholesterol; HDL-C: high-density lipoprotein cholesterol

According to Fig. 1, changes in TyG-index levels from the first to fifth quintiles showed significant increasing trends (P-value = 0.002 and < 0.001) for incident CVD and CHD, respectively. Compared to the reference, adjusted HRs (95% CI) for incident CVD for the second, third, fourth and fifth quintiles were 1.15 (0.89–1.49), 1.28 (0.99–1.65), 1.22 (0.94–1.58), and 1.61 (1.23–2.11); and the corresponding HRs for incident CHD were 1.25 (0.93–1.67), 1.49 (1.12–1.98), 1.34 (1.01–1.80), and 1.84 (1.37–2.48), respectively. According to multivariate Cox regression analysis for incident CVD, aging, abdominal adiposity, smoking, family history of CVD, T2D, hypertension, and a higher value of LDL-C increased the risk of CVD events; whereas, BMI and female gender were associated with lower risk. Similar results are shown in Fig. 1 for incident CHD; however, a significant association was found between higher values of HLD-C and lower risk for incident CHD. Regarding the inverse association of BMI for incident CVD/CHD in our data analysis, after excluding WC and obesity mediators (including T2D, hypertension, LDL-C, HDL-C, and TyG-index) [32], higher BMI was significantly associated with higher risk of CVD/CHD events [HR: 1.04, 95% CI (1.03–1.06)]; the risks however, reached the null after further adjustment for obesity mediators. After further adjustment for WC, higher BMI was associated with lower risk of events (Additional file 1: Table S1 and S2).

### TyG-index as a continuous variable

Table 2 shows the association between 1SD increase in TyG-index and incident CVD based on multivariate Cox proportional hazard analyses among males, females and total population. Accordingly, 1SD increase in TyG-index for incident CVD were 1.11 (1.01-1.22), 1.23 (1.09-1.39), and 1.16 (1.07-1.25), respectively; the corresponding values for incident CHD were 1.16 (1.05-1.29), 1.23 (1.07-1.39), and 1.19 (1.10-1.29), (Table 3).

**Table 2:** Multivariable-adjusted hazard ratios of incident CVD according to the 1-SD increase in TyG-index, stratified by gender: Tehran Lipid and Glucose Study

Variables	Male (n = 4154)		Female (n = 3367)		Total (n = 7521)	
	HR (95% CI)	P value	HR (95% CI)	P value	HR (95% CI)	P value
TyG-index	1.11 (1.01–1.22)	0.040	1.23 (1.09–1.39)	0.001	1.16 (1.07–1.25)	< 0.001
Age (years)	1.06 (1.05–1.06)	< 0.001	1.05 (1.04–1.06)	< 0.001	1.05 (1.05–1.06)	< 0.001
WC (cm)	1.01 (1.00-1.03)	0.105	1.02 (1.00-1.03)	0.022	1.02 (1.01–1.03)	0.004
BMI (kg/m^2^)	0.98 (0.93–1.02)	0.261	0.98 (0.94–1.01)	0.155	0.97 (0.95–1.00)	0.052
Education (years)						
< 6	Reference		Reference		Reference	
6–12	1.11 (0.92–1.35)	0.289	0.81 (0.61–1.07)	0.135	0.99 (0.85–1.16)	0.922
≥ 12	0.98 (0.75–1.29)	0.903	0.59 (0.30–1.16)	0.124	0.88 (0.69–1.12)	0.299
Smoking						
Never	Reference		Reference		Reference	
Past	1.37 (1.12–1.69)	0.003	1.01 (0.63–1.61)	0.983	1.31 (1.09–1.57)	0.005
Current	1.67 (1.39–2.02)	< 0.001	1.62 (1.07–2.46)	0.023	1.67 (1.41–1.97)	< 0.001
Low physical activity	1.05 (0.88–1.25)	0.608	1.11 (0.90–1.38)	0.333	1.07 (0.94–1.23)	0.301
FH-CVD	1.37 (1.10–1.70)	0.004	1.58 (1.28–1.96)	< 0.001	1.47 (1.26–1.71)	< 0.001
T2D	1.73 (1.39–2.15)	< 0.001	1.80 (1.41–2.30)	< 0.001	1.78 (1.51–2.09)	< 0.001
Hypertension	1.60 (1.34–1.91)	< 0.001	1.65 (1.34–2.04)	< 0.001	1.64 (1.43–1.87)	< 0.001
Lipid drug	0.97 (0.63–1.50)	0.895	1.20 (0.90–1.61)	0.208	1.12 (0.88–1.42)	0.346
LDL-C (mmol/L)	1.37 (1.25–1.49)	< 0.001	1.18 (1.07–1.29)	0.001	1.28 (1.21–1.37)	< 0.001
HDL-C (mmol/L)	0.76 (0.53–1.10)	0.146	0.87 (0.60–1.25)	0.439	0.82 (0.63–1.06)	0.133
Gender (female)	–	–	–	–	0.57 (0.49–0.66)	<0.001

*CVD* cardiovascular disease, *SD* standard deviation, *TyG-index* triglyceride glucose-index, *HR* hazard ratio, *CI* confidence interval, *WC* waist circumference, *BMI* body mass index, *FH-CVD* family history of CVD, *T2D* type 2 diabetes, *LDL-C* low density lipoprotein cholesterol, *HDL-C* high density lipoprotein cholesterol

**Table 3:** Multivariable-adjusted hazard ratios of incident CHD according to the 1-SD increase in TyG-index, stratified by genders: Tehran Lipid and Glucose Study

Variables	Male (n = 4154)		Female (n = 3366)		Total (n = 7520)	
	HR (95% CI)	P value	HR (95% CI)	P value	HR (95% CI)	P value
TyG-index	1.16 (1.05–1.29)	0.005	1.23 (1.07–1.39)	0.003	1.19 (1.10–1.29)	< 0.001
Age (years)	1.05 (1.04–1.06)	< 0.001	1.04 (1.03–1.05)	< 0.001	1.05 (1.04–1.05)	< 0.001
WC (cm)	1.02 (1.00–1.04)	0.036	1.02 (1.00–1.03)	0.066	1.02 (1.01–1.03)	0.004
BMI (kg/m^2^)	0.96 (0.92–1.01)	0.100	0.97 (0.94–1.01)	0.162	0.97 (0.94–1.00)	0.020
Education (years)						
< 6	Reference		Reference		Reference	
6–12	1.21 (0.98-1.49)	0.075	0.81 (0.60–1.09)	0.161	1.04 (0.88–1.23)	0.629
≥ 12	1.02 (0.76-1.36)	0.904	0.57 (0.27–1.17)	0.122	0.88 (0.68–1.15)	0.343
Smoking						
Never	Reference		Reference		Reference	
Past	1.39 (1.11–1.75)	0.004	1.08 (0.65–1.80)	0.768	1.34 (1.09–1.64)	0.005
Current	1.67 (1.37–2.04)	< 0.001	1.68 (1.08–2.59)	0.020	1.68 (1.41–2.01)	< 0.001
Low physical activity	1.09 (0.90–1.32)	0.382	1.06 (0.84–1.33)	0.650	1.08 (0.93–1.25)	0.325
FH-CVD	1.39 (1.10–1.74)	0.005	1.63 (1.30–2.06)	< 0.001	1.51 (1.28–1.77)	< 0.001
T2D	1.53 (1.20–1.95)	0.001	2.01 (1.54–2.61)	< 0.001	1.74 (1.46–2.08)	< 0.001
Hypertension	1.45 (1.19–1.76)	< 0.001	1.73 (1.37–2.17)	< 0.001	1.57 (1.35–1.81)	< 0.001
Lipid drug	0.91 (0.56–1.48)	0.696	1.24 (0.91–1.69)	0.171	1.12 (0.87–1.45)	0.380
LDL-C (mmol/L)	1.37 (1.25–1.50)	< 0.001	1.21 (1.09–1.33)	< 0.001	1.30 (1.22–1.39)	< 0.001
HDL-C (mmol/L)	0.71 (0.48–1.06)	0.097	0.69 (0.46–1.03)	0.072	0.72 (0.54–0.96)	0.023
Gender (female)	–	–	–	–	0.60 (0.50–0.71)	< 0.001

*CHD* coronary heart disease, *SD* standard deviation, *TyG-index* triglyceride glucose-index, *HR* hazard ratio, *CI* confidence interval, *WC* waist circumference, *BMI* body mass index, *FH-CVD* family history of cardiovascular disease, *T2D* type 2 diabetes, *LDL-C* low density lipoprotein cholesterol, *HDL-C* high density lipoprotein cholesterol

### TyG-index cut off points

**Fig. 2:**
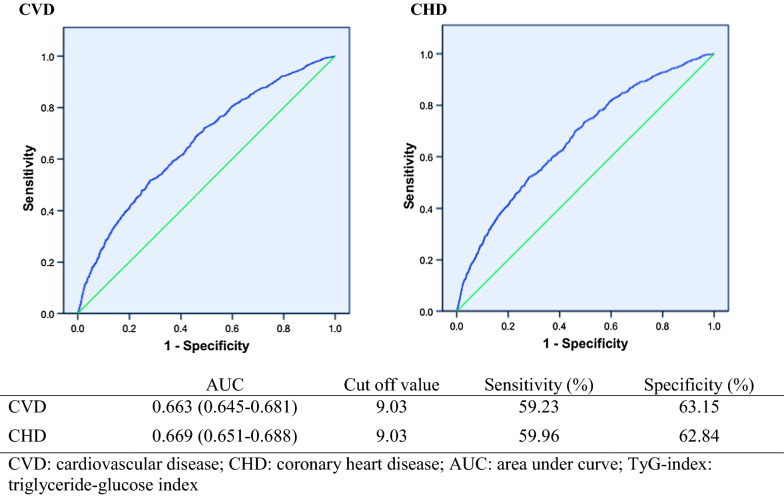
Receiver operative characteristic curves and cut off values of TyG-index for incident CVD/CHD

Among population with complete follow-up (n = 6463), area under curve (AUC) of the TyG-index for incidence CVD/CHD were 0.633, 95% CI (0.645–0.681) and 0.669, 95% CI (0.651–0.688), respectively (Fig. 2). The cut-off points for TyG-index for incident CVD/CHD among the whole population are also shown in Fig. 2. Cut-off value for TyG-index for incident CVD was calculated to be 9.03 with 59.23% sensitivity and 63.15% specificity; the corresponding values incident CHD was also 9.03 with 59.96% sensitivity and 62.84% specificity, respectively.

### Sensitivity analysis

**Fig. 3:**
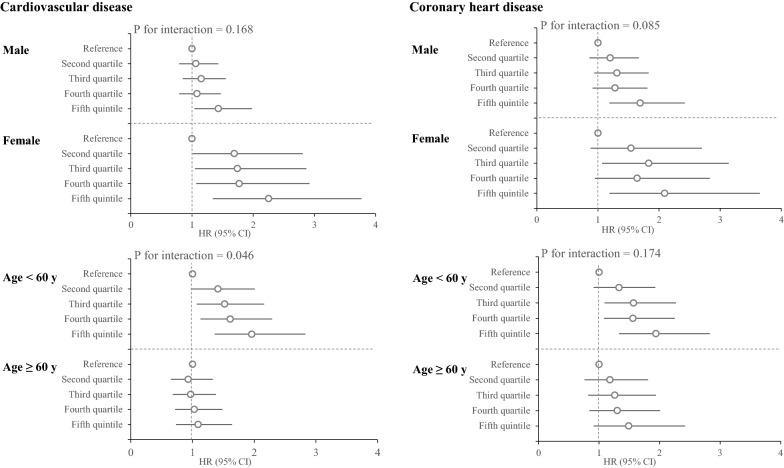
Association between TyG-index quintiles and incident CVD/CHD stratified by gender and age. Stratified analyses were adjusted for WC, BMI, education, smoking status, physical activity, family history of CVD, T2D, hypertension, lowering lipid drugs, LDL-C, and HDL-C plus “age” in gender-stratified and “gender” in age-stratified analyses. TyG-index: Triglyceride-Glucose index; CVD: cardiovascular disease; CHD: coronary heart disease; WC: waist circumference; BMI: body mass index; T2D: type 2 diabetes; LDL-C: low density lipoprotein cholesterol; HDLC: high density lipoprotein cholesterol; HR: hazard ratio; CI: confidence interval

To assess the robustness of our findings, we also performed sensitivity analyses according to gender, and among age groups, separately, in the multivariate adjusted models (Fig. 3). Although no interaction was found between gender and TyG-index for CVD/CHD in multivariate analysis (Ps for interaction > 0.085), the significant trend of TyG-index was observed only among females for incident CVD (P = 0.035). HRs and 95% CIs from the second to fifth quintile were 1.69 (1.01–2.81), 1.74 (1.05–2.87), 1.77 (1.07–2.92), and 2.25 (1.34–3.77), respectively. The corresponding values for incident CHD were 1.54 (0.88–2.70), 1.83 (1.07–3.14), 1.64 (0.95–2.83), and 2.09 (1.19–3.65), respectively.

Age-stratified analyses showed a significant trend of TyG-index (Ps for trend ≤ 0.010) for incident CVD/CHD among the younger age group (< 60 years); however, risks reached the null in subjects who were 60 years or older. The adjusted HR of TyG-index from the second to fifth quintile, among population aged < 60 years for incident CVD were 1.41 (0.98–2.01), 1.52 (1.07–2.16), 1.61 (1.13–2.29), 1.96 (1.36–2.83)] corresponding values for incident CHD were 1.33 (0.91–1.93), 1.57 (1.09–2.27), 1.56 (1.08–2.25), 1.94 (1.33–2.83), respectively (Fig. 3).

### Added value of TyG-index to FRS

The predictive value of TyG-index for CVD events was assessed using Framingham risk score components among population aged < 60 years. As shown in Table 4, in the model including both FRS and TyG-index, the HR of these variables for incident CVD was 1.06 (1.05–1.06) and 1.42 (1.26–1.60), respectively. In addition, the C-index of the FRS model did not change after addition of the TyG-index [0.79 (0.77–0.81) and 0.77 (0.75–0.79), respectively]. IDI statistics did not show any improvement in the predictive ability of the FRS model [0.001 − 0.004 to 0.007)].

**Table 4:** Clinical performance of the model to predict 10-year cardiovascular risk containing FRS and FRS + TyG-index models in the Population < 60 years (n = 6175): Tehran Lipid and Glucose Study

	FRS model* HR (95% CI)	P-value	FRS + TyG-index HR (95% CI)	P-value
Model components				
FRS	1.07 (1.06–1.07)	< 0.001	1.06 (1.05–1.06)	< 0.001
TyG-index	–		1.42 (1.26–1.60)	< 0.001
Model predictive performance indexes				
C-index	0.789 (0.773-0.805)	<0.001	0.771 (0.752–0.789)	< 0.001
IDI	–	–	0.001 (−0.004 to 0.007)	0.673

*FRS* Framingham risk score, *TyG-index* Triglyceride-Glucose index, *HR* hazard ratio, *CI* confidence interval, *C-index* Harrell’s concordance statistic, *IDI* integrated discrimination improvement

*FRS model: Framingham risk score model including age, gender, total cholesterol, high density lipoprotein cholesterol, systolic blood pressure, anti-hypertensive drugs, smoking, and type 2 diabetes

## Discussion

In the present study, we found that higher plasma TyG-index level, either as a continuous or categorical variable was associated with increased risk for CVD/CHD incidence, independent of other traditional CVD risk factors. The TyG-index cut-off values for both incident CVD and CHD were the same (9.03) with sensitivity and specificity of around 60%. Moreover, a significant association between TyG-index and CVD/CHD was mainly observed among younger adults; although no improvement was observed in prediction of CVD beyond that achieved by FRS.

TyG-index was previously introduced as a surrogate marker of IR [6]. Previous studies have reported an association between IR and important CVD risk factors such as T2D and hypertension [33, 34]. IR is also considered to be strongly correlated with the risk of incident CVD [35, 36]. Moreover, we previously showed that the presence of IR among the non-hypertensive population was associated with CVD events [37]. There is a plausible biological mechanism underlying the association between TyG-index and incident CVD through the strong relationship between IR and endothelial dysfunction [38]. This mostly includes inflammation and functional impairment in the endothelium of blood vessels [39–41] leading to atherosclerosis and CVD [42]. However, the precise mechanism underlying the association between IR and CVD remains unclear. Therefore, considering the difficulty in direct estimation of insulin action and due to the absence of a standard insulin assay, TyG-index as a surrogate of IR may be associated with incident CVD.

### TyG-index and Cardiovascular Outcomes

The association between TyG-index and CVD has also been supported by cross-sectional [43–48] and case–control studies [49, 50]. Moreover, to the best of our knowledge, there have been three prospective [13–15] and three retrospective [16, 51, 52] studies examining the association between TyG-index and cardiovascular outcomes among participants without baseline CVD.

Recently in a retrospective cohort study, Li et al. [16] evaluated the risk of TyG-index on incident CVD among the elderly population. Compared to the lowest quartile, the highest quartile was associated with a higher risk for CVD in the multivariate-adjusted model. Furthermore, Park et al. [51] reported that TyG-index is an independent predictor of coronary artery calcification progression. Moreover, in a study conducted among South Korean adults, TyG-index was reported as an independent predictor of coronary artery calcification (CAC) progression; this issue was more prominent among adults without heavy CAC at baseline [52].

Among non-obese and relatively healthy American white males, TyG-index ≥ 15 was associated with higher risk of CVD/CHD mortality; however, the risk of TyG-index disappeared after further adjustment for non-HDL-C [13]. Sánchez Iñigo et al.  [14] reported that TyG-index was significantly associated with a higher risk for incident CVD i.e. those at the fourth and fifth quintiles had 1.52 and 2.32 higher risk for CVD events among the European population. Meanwhile Salazar et al. [15] conducted a study among Argentinians aged 15–80 years, reporting that TyG-index as a continuous variable was associated with 46% higher risk for CVD in multivariate analysis; however, the risk for this measure did not reach a significant level when TyG-index was treated as a categorical variable. In our data analysis, the fifth quintile of TyG-index had about 60 and 80% higher risk for incident CVD and CHD in the multivariate analyses, respectively. We also found that a 1-SD increase in TyG-index was associated with about 20% higher risk for CVD events.

As expected, modifiable traditional risk factors including abdominal obesity, smoking, hypertension, T2D, LDL-C remained significantly associated with incident CVD/CHD; however, BMI and female gender were inversely associated with the incidence of both events in multivariate analysis. In the current study, in the absence of obesity mediators, BMI showed a significant risk for CVD events whereas together they were not associated with either event. Finally, when we adjusted WC in the analysis (an index of abdominal obesity), BMI changed direction, becoming inversely associated with incident CVD/CHD. This finding might be due to the fact that BMI is a reflection of lean mass for individuals with the same WC, whereas WC is a reflection of abdominal fat content for individuals with the same BMI [53]. Thus, we supposed that in our data analysis, BMI value when adjusted for WC might indicate the level of muscle mass. In line with our results, BMI was inversely associated with all-cause mortality in the Canadian population [54]. Regarding the lower risk of female gender, an issue addressed in our previous articles [28, 55], this might be related to baseline socioeconomic differences among genders. Our study was initiated in 1999–2000 in a developing country where females share in public sector employment was registered at 30.33 percent in 1999 and most females worked in households, whereas males were mostly occupied with outdoor jobs [56]. Hence, males were more in contact with chemicals and non-chemical environmental factors such as temperature, noise exposure, physiological stress, and air pollution which potentially amplify its association with cardiovascular events [57].

### TyG-index cut off point

Considering TyG-index cut-off points, cohort studies conducted among Asian populations examined this issue for identifying the development of T2D. In a study conducted among Korean adults, the optimal cut-off value for TyG-index was 8.8, with the area under the ROC curve amounting to 0.751 (95% CI 0.704–0.799) [58]. Another study conducted in Korea reported a cut-off point of 8.86 for TyG-index (sensitivity of 56.01 and specificity of 57.11%) for predicting T2D among males, with the corresponding value for females being 8.52 (sensitivity of 67.25 and specificity of 55.85%) [59]. The cut-off values of TyG-index for prevalent hypertension in a cross-sectional study among the Chinese population were reported as 9.04 (sensitivity of 52.58 and specificity of 72.27%) in males and 8.59 (sensitivity of 76.07 and specificity of 46.69%) in females [60]. Mao et al. estimated a cut-off value of 8.56 with sensitivity of 87.2 and specificity of 35.3% for TyG-index for recurrent cardiovascular outcomes, which was assessed by ROC curve analysis [61]. Hence, our derived corresponding cut-off points of TyG-index for incident CVD and CHD among Iranian population were comparable to those values for detecting prevalent hypertension and were higher than those reported for predicting incident T2D and recurrent CVD among the East Asian population.

### Added value of TyG-index to FRS

We previously validated and examined the clinical usefulness of FRS in prediction of CVD events among Tehranian population (30–74 years) [19]. In the current study, we fitted predictive models for incident CVD among younger adults using FRS variables, with and without further addition of the TyG-index. In our data analyses, no improvement was observed in the prediction of CVD risk after further addition of the TyG-index to the Framingham predictors. In this regard, Sanchez et al. [14] plotted AUC of the ROC curves for the Framingham model and Framingham + TyG-index model. The added predictive value of the model including TyG-index was seen only among participants at 10 to 20% of 10-year cardiovascular risk.

### Strengths and limitations

There are some strengths for this study that should be mentioned. This is the first long-term population-based prospective study conducted in the MENA region in a population with a high burden of CVD events. Second, we assessed the precise value of CVD risk factors rather than relying on self-reported data. Third, the statistical power of our study according to the effect size of the fifth quintile and a 1-SD increase TyG-index in the multivariate analysis were 96% and 33%, respectively.

There are a few limitations that must also be noted. First, previous studies reported that variation of TyG-index during the follow-up time could modify the relationship between this index and CVD [61]. We used only the baseline values of TyG-index and other risk factors and we did not consider changes in these parameters over the follow-up time. Second, this is a population-based study conducted among participants resident in the metropolitan city of Tehran; hence, our findings may not be generalizable to other populations. Third, we did not record nutritional habits or energy intake which might affected TG levels. However, we did adjust for other related confounders such as BMI and lipid measures.

## Conclusion

During more than a decade of follow-up we found that TyG-index (as a surrogate for IR) was a significant risk factor for incident CVD/CHD, whether as a continuous or categorical variable; an issue that was more prominent among the younger population. In addition, we recommend a cut-off point of 9 for TyG-index to identify Iranian adults who are at risk for future CVD/CHD. Finally, adding TyG-index to FRS does not provide greater risk prediction for CVD.

## Supplementary information

**Additional file 1: Table S1.** Adjusted hazard ratio of BMI for incident CVD according to Cox proportional hazard models: Tehran Lipid and Glucose Study. **Tables S2.** Adjusted hazard ratio of BMI for incident CHD according to Cox proportional hazard models: Tehran Lipid and Glucose Study.

## Data Availability

The datasets used and/or analyzed during the current study are available from the corresponding author on reasonable request.

## Abbreviations

CVD: Cardiovascular diseases
MENA: Middle East and North Africa
IR: Insulin resistance
T2D: Type 2 diabetes
TyG-index: Triglyceride-glucose index
TG: Triglyceride
FPG: Fasting plasma glucose
CHD: Coronary heart disease
TLGS: Tehran Lipid and Glucose Study
LRC: Lipid Research Clinic
MAQ: Modifiable Activity Questionnaire
BMI: Body mass index
SBP: Systolic blood pressure
DBP: Diastolic blood pressure
TC: Total cholesterol
HDL-C: High-density lipoprotein cholesterol
2hPG: 2 h plasma glucose
LDL-C: Low-density lipoprotein cholesterol
MI: Myocardial infarction
ECG: Electrocardiogram
MET: Metabolic equivalent task
SD: Standard deviation
HR: Hazard ratio
CI: Confidence interval
WC: Waist circumference
ROC: Receiver operation characteristic
