# Supplementary material for: The impact of triglyceride-glucose index on incident cardiovascular events during 16 years of follow-up: Tehran Lipid and Glucose Study
Source: Cardiovasc Diabetol. 2020 Sep 29;19:155. doi: 10.1186/s12933-020-01121-5 (PMC7526412; doi:10.1186/s12933-020-01121-5)
Supplement: Supplementary file 1 — Additional file 1: Table S1. Adjusted hazard ratio of BMI for incident CVD according to Cox proportional hazard models: Tehran Lipid and Glucose Study. Tables S2. Adjusted hazard ratio of BMI for incident CHD according to Cox proportional hazard models: Tehran Lipid and Glucose Study. [file 12933_2020_1121_MOESM1_ESM.docx]

| **Supplemental Table 1.** Adjusted hazard ratio of BMI for incident CVD according to cox proportional hazard models: Tehran Lipid and Glucose Study | | | | | | | | | | |
| --- | --- | --- | --- | --- | --- | --- | --- | --- | --- | --- |
|  | |  | Model I |  |  | Model II |  |  | Model III |  |
| Variables | |  | HR (95% CI) | P value |  | HR (95% CI) | P value |  | HR (95% CI) | P value |
| BMI (kg/m^2^) | |  | 1.04 (1.03-1.06) | <0.001 |  | 1.00 (0.99-1.02) | 0.608 |  | 0.97 (0.95-1.00) | 0.036 |
| Age (year) | |  | 1.07 (1.06-1.07) | <0.001 |  | 1.06 (1.05-1.06) | <0.001 |  | 1.05 (1.05-1.06) | <0.001 |
| Gender (female) | |  | 0.59 (0.51-0.68) | <0.001 |  | 0.54 (0.46-0.63) | <0.001 |  | 0.57 (0.49-0.67) | <0.001 |
| Education (year) | |  |  |  |  |  |  |  |  |  |
|  | < 6 |  | Reference |  |  | Reference |  |  | Reference |  |
|  | 6-12 |  | 0.97 (0.83-1.13) | 0.658 |  | 0.98 (0.84-1.15) | 0.837 |  | 0.99 (0.85-1.17) | 0.978 |
|  | ≥ 12 |  | 0.86 (0.67-1.10) | 0.220 |  | 0.87 (0.68-1.11) | 0.259 |  | 0.88 (0.69-1.12) | 0.290 |
| Smoking | |  |  |  |  |  |  |  |  |  |
|  | Never |  | Reference |  |  | Reference |  |  | Reference |  |
|  | Past |  | 1.29 (1.07-1.56) | 0.007 |  | 1.32 (1.10-1.59) | 0.003 |  | 1.31 (1.09-1.58) | 0.004 |
|  | Current |  | 1.61 (1.37-1.90) | <0.001 |  | 1.69 (1.43-2.00) | <0.001 |  | 1.68 (1.42-1.98) | <0.001 |
| Low physical activity | |  | 1.10 (0.96-1.26) | 0.165 |  | 1.08 (0.94-1.24) | 0.265 |  | 1.08 (0.94-1.24) | 0.276 |
| FH-CVD | |  | 1.52 (1.30-1.76) | <0.001 |  | 1.46 (1.26-1.70) | <0.001 |  | 1.46 (1.26-1.70) | <0.001 |
| T2D | |  | - | - |  | 1.84 (1.58-2.15) | <0.001 |  | 1.81 (1.55-2.11) | <0.001 |
| Hypertension | |  | - | - |  | 1.66 (1.45-1.90) | <0.001 |  | 1.64 (1.44-1.88) | <0.001 |
| Lipid drug | |  | 1.72 (1.36-2.16) | <0.001 |  | 1.13 (0.89-1.43) | 0.318 |  | 1.12 (0.88-1.42) | 0.362 |
| LDL-C (mmol/L) | |  | - | - |  | 1.29 (1.21-1.37) | <0.001 |  | 1.29 (1.21-1.37) | <0.001 |
| HDL-C (mmol/L) | |  | - | - |  | 0.80 (0.62-1.03) | 0.083 |  | 0.82 (0.63-1.06) | 0.130 |
| TyG-index | |  |  |  |  |  |  |  |  |  |
|  | First quintile |  | - | - |  | Reference |  |  | Reference |  |
|  | Second quintile |  | - | - |  | 1.18 (0.91-1.53) | 0.206 |  | 1.15 (0.89-1.49) | 0.277 |
|  | Third quintile |  | - | - |  | 1.32 (1.03-1.70) | 0.031 |  | 1.28 (0.99-1.65) | 0.058 |
|  | Fourth quintile |  | - | - |  | 1.27 (0.98-1.64) | 0.071 |  | 1.22 (0.94-1.58) | 0.130 |
|  | Fifth quintile |  |  |  |  | 1.69 (1.29-2.20) | <0.001 |  | 1.61 (1.23-2.11) | <0.001 |
| WC (cm) | |  | - | - |  | - | - |  | 1.02 (1.01-1.03) | 0.004 |
| BMI: body mass index; CVD: cardiovascular disease; HR: hazard ratio; CI: confidence interval; FH-CVD: family history of CVD; T2D: type 2 diabetes; LDL-C: low density lipoprotein cholesterol; HDL-C: high density lipoprotein cholesterol; TyG-index: triglyceride glucose-index; WC: waist circumference. Model I: Adjusted for BMI, age, gender, education, smoking, physical activity, FH-CVD, and lipid drug; Model II: Model I + T2D, hypertension, LDL-C, HDL-C, and TyG-index; Model III: model II+ WC | | | | | | | | | | |

| **Supplemental Table 2.** Adjusted hazard ratio of BMI for incident CHD according to cox proportional hazard models: Tehran Lipid and Glucose Study | | | | | | | | | | |
| --- | --- | --- | --- | --- | --- | --- | --- | --- | --- | --- |
|  | |  | Model I |  |  | Model II |  |  | Model III |  |
| Variables | |  | HR (95% CI) | P value |  | HR (95% CI) | P value |  | HR (95% CI) | P value |
| BMI (kg/m^2^) | |  | 1.04 (1.03-1.06) | <0.001 |  | 1.00 (0.98-1.02) | 0.894 |  | 0.96 (0.94-0.99) | 0.013 |
| Age (year) | |  | 1.06 (1.05-1.07) | <0.001 |  | 1.05 (1.04-1.05) | <0.001 |  | 1.05 (1.04-1.05) | <0.001 |
| Gender (female) | |  | 0.59 (0.50-0.69) | <0.001 |  | 0.57 (0.48-0.67) | <0.001 |  | 0.60 (0.51-0.71) | <0.001 |
| Education (year) | |  |  |  |  |  |  |  |  |  |
|  | < 6 |  | Reference |  |  | Reference |  |  | Reference |  |
|  | 6-12 |  | 1.00 (0.85-1.19) | 0.964 |  | 1.02 (0.87-1.21) | 0.733 |  | 1.05 (0.88-1.24) | 0.596 |
|  | ≥ 12 |  | 0.86 (0.66-1.11) | 0.237 |  | 0.87 (0.67-1.13) | 0.288 |  | 0.88 (0.68-1.14) | 0.325 |
| Smoking | |  |  |  |  |  |  |  |  |  |
|  | Never |  | Reference |  |  | Reference |  |  | Reference |  |
|  | Past |  | 1.33 (1.09-1.63) | 0.006 |  | 1.34 (1.10-1.65) | 0.003 |  | 1.35 (1.10-1.65) | 0.004 |
|  | Current |  | 1.67 (1.40-1.99) | <0.001 |  | 1.72 (1.44-2.05) | <0.001 |  | 1.70 (1.42-2.03) | <0.001 |
| Low physical activity | |  | 1.11 (0.96-1.28) | 0.178 |  | 1.09 (0.94-1.26) | 0.298 |  | 1.08 (0.93-1.25) | 0.307 |
| FH-CVD | |  | 1.55 (1.32-1.82) | <0.001 |  | 1.50 (1.28-1.76) | <0.001 |  | 1.50 (1.28-1.76) | <0.001 |
| T2D | |  | - | - |  | 1.87 (1.59-2.21) | <0.001 |  | 1.81 (1.52-2.14) | <0.001 |
| Hypertension | |  | - | - |  | 1.59 (1.37-1.83) | <0.001 |  | 1.58 (1.36-1.83) | <0.001 |
| Lipid drug | |  | 1.76 (1.37-2.26) | <0.001 |  | 1.15 (0.89-1.49) | 0.345 |  | 1.12 (0.87-1.45) | 0.387 |
| LDL-C (mmol/L) | |  | - | - |  | 1.31 (1.22-1.40) | <0.001 |  | 1.30 (1.22-1.39) | <0.001 |
| HDL-C (mmol/L) | |  | - | - |  | 0.69 (0.52-0.92) | 0.013 |  | 0.72 (0.54-0.95) | 0.022 |
| TyG-index | |  |  |  |  |  |  |  |  |  |
|  | First quintile |  | - | - |  | Reference |  |  | Reference |  |
|  | Second quintile |  | - | - |  | 1.28 (0.96-1.71) | 0.099 |  | 1.25 (0.93-1.67) | 0.136 |
|  | Third quintile |  | - | - |  | 1.54 (1.16-2.04) | 0.003 |  | 1.49 (1.12-1.98) | 0.006 |
|  | Fourth quintile |  | - | - |  | 1.40 (1.05-1.87) | 0.023 |  | 1.34 (1.01-1.80) | 0.046 |
|  | Fifth quintile |  |  |  |  | 1.93 (1.43-2.60) | <0.001 |  | 1.84 (1.37-2.48) | <0.001 |
| WC (cm) | |  | - | - |  | - | - |  | 1.02 (1.01-1.03) | 0.003 |
| BMI: body mass index; CHD: coronary heart disease; HR: hazard ratio; CI: confidence interval; FH-CVD: family history of cardiovascular disease; T2D: type 2 diabetes; LDL-C: low density lipoprotein cholesterol; HDL-C: high density lipoprotein cholesterol; TyG-index: triglyceride glucose-index; WC: waist circumference. Model I: Adjusted for BMI, age, gender, education, smoking, physical activity, FH-CVD, and lipid drug; Model II: Model I + T2D, hypertension, LDL-C, HDL-C, and TyG-index; Model III: model II+ WC | | | | | | | | | | |
